# COVID-19 Misinformation Trends in Australia: Prospective Longitudinal National Survey

**DOI:** 10.2196/23805

**Published:** 2021-01-07

**Authors:** Kristen Pickles, Erin Cvejic, Brooke Nickel, Tessa Copp, Carissa Bonner, Julie Leask, Julie Ayre, Carys Batcup, Samuel Cornell, Thomas Dakin, Rachael H Dodd, Jennifer M J Isautier, Kirsten J McCaffery

**Affiliations:** 1 The University of Sydney Sydney Australia

**Keywords:** COVID-19, coronavirus, misinformation, infodemic, myths, conspiracy, digital health, literacy, social media, trust

## Abstract

**Background:**

Misinformation about COVID-19 is common and has been spreading rapidly across the globe through social media platforms and other information systems. Understanding what the public knows about COVID-19 and identifying beliefs based on misinformation can help shape effective public health communications to ensure efforts to reduce viral transmission are not undermined.

**Objective:**

This study aimed to investigate the prevalence and factors associated with COVID-19 misinformation in Australia and their changes over time.

**Methods:**

This prospective, longitudinal national survey was completed by adults (18 years and above) across April (n=4362), May (n=1882), and June (n=1369) 2020.

**Results:**

Stronger agreement with misinformation was associated with younger age, male gender, lower education level, and language other than English spoken at home (*P*<.01 for all). After controlling for these variables, misinformation beliefs were significantly associated (*P*<.001) with lower levels of digital health literacy, perceived threat of COVID-19, confidence in government, and trust in scientific institutions. Analyses of specific government-identified misinformation revealed 3 clusters: prevention (associated with male gender and younger age), causation (associated with lower education level and greater social disadvantage), and cure (associated with younger age). Lower institutional trust and greater rejection of official government accounts were associated with stronger agreement with COVID-19 misinformation.

**Conclusions:**

The findings of this study highlight important gaps in communication effectiveness, which must be addressed to ensure effective COVID-19 prevention.

## Introduction

False, misleading, or inaccurate health information can pose a serious risk to public health and public action [1]. Misinformation about COVID-19 is common and has spread rapidly across the globe through social media platforms and other information systems [2-5]. In February 2020, the World Health Organization’s Director-General declared the global “overabundance” of COVID-19 information an “infodemic” [6]. The term “misinfodemic” has since been coined to capture the corresponding increase in misinformation surrounding the virus [7].

Misinformation, which is typically compelling, persuasive, and emotive, spreads on social media platforms significantly farther, faster, deeper, and more broadly than factual information [8]. This is particularly true within tight-knit communities, as has been observed with the spread of vaccine misinformation among some communities in the United States, Sweden, and the Netherlands [9-12]. Common COVID-19 beliefs circulating in mainstream media include framing the pandemic as a leaked bioweapon, a consequence of 5G wireless technology, a political hoax, and that the pandemic has been made up by governments to control people. Others detail ineffective measures that individuals can take to prevent or treat the disease, such as exposing themselves to sunlight or taking vitamin C [13].

Misinformation can undermine public health efforts by shaping beliefs and attitudes, particularly if encountered within a social network, and reinforcing pre-existing values and positions [14]. Importantly, lower perceived risk or perceived efficacy of prevention behaviors and altered perception of social norms might influence individuals’ willingness to follow recommendations such as voluntary testing, isolation and, potentially, vaccination [15].

Understanding what the public knows about COVID-19 and identifying beliefs based on misinformation can help shape effective public health communication to ensure effort to reduce its impact, such as debunking [16].

This paper uses data from a longitudinal cohort study of the Australian public. Our aims were to: (1) investigate the prevalence of COVID-19 misinformation beliefs in the study sample; (2) examine whether any demographic, psychosocial, and cognitive factors are associated with these beliefs; and (3) investigate how these misinformation beliefs change over time.

## Methods

### Data Collection

The data used in this study are from a prospective, longitudinal, national survey in Australia that aimed to evaluate variation in the public’s understanding, attitudes, and implementation of COVID-19 health advice during the first lockdown period in 2020 [17,18]. A total of 4362 participants were recruited between April 17 and 24, 2020; these participants completed the baseline survey (Round 1). This survey was administered 1 month after the first measures of prevention (physical distancing and quarantine) were introduced in Australia when an increasing number of COVID-19 cases were being reported. A subset (n=3214) of this sample was invited for a longitudinal follow-up to assess changes in attitudes, beliefs, and behavior over the course of the pandemic. Of the 3214 participants, 1882 (58.5%) were invited for the Round 2 survey, which was administered from May 8 to 15, 2020 (ie, 3 weeks after the baseline or Round 1). Round 3 survey was administered to 1369 of the 3214 participants (43%) from June 5 to 12, 2020 (ie, approximately 6 weeks after the baseline survey), when restrictions in Australia showed signs of easing, and the number of new COVID-19 cases and reported community transmission had drastically reduced. Round 3 survey was administered prior to the resurgence of COVID-19 cases in some areas of Australia.

### Recruitment

Participants were recruited via advertisements on social media (ie, Facebook and Instagram) and by a market research company (Dynata). We used 2 different methods for recruitment with the aim of achieving a more diverse sample. Only those participants who were recruited via social media were invited for the longitudinal follow-up.

Dynata is a market research company with access to a database of 600,000 members in Australia who are willing to be involved in online research studies. Dynata invites members to participate in a certain research study only when they meet the study’s eligibility criteria. For instance, only participants who met the following eligibility criteria were included in this study: adults (ie, age 18 years or older), currently living in Australia, and ability to read and understand English.

Participants recruited via Dynata received points for completing the survey; these points could then be redeemed against gift vouchers, donations to charities, or cash. Participants recruited via social media were given the opportunity to enter into a prize draw for the chance to win one of ten $20 gift cards upon completion of each survey round.

### Ethics Approval

Ethics approval was obtained from the Human Research Ethics Committee of The University of Sydney (2020/212). Participants were informed about the purpose of the study, confidentiality, and risks and benefits of participation at the beginning of the survey. Completion and submission of the online questionnaire were considered as evidence of consent.

### Measures

The survey was built and administered using Qualtrics (SAP SE), an online survey platform, and it was piloted within the health literacy lab. Survey items included in each round were modified from the national longitudinal study [17] to reflect psychological, behavioral, and knowledge factors considered most relevant at that stage of restrictions. Relevant measures for this study are detailed in Table 1. Age, gender, education, language other than English (LOTE) spoken at home, and socioeconomic status were assessed in Round 1, as detailed in our previous study [17].

**Table 1:** Measures evaluated in this study.

Item		Description and reference (if applicable)	April	May	June		
**Main outcomes**							
	**COVID-19–related misinformation beliefs^a^**						
		Data on the effectiveness of vaccines is often made up^b^.	✓				
		Herd immunity would be beneficial for COVID-19 and this fact is covered up.	✓	✓			
		The threat of COVID-19 is greatly exaggerated.	✓	✓	✓		
		Government restrictions are stronger than is needed.	✓	✓	✓		
	**Specific COVID-19 misinformation^c^**						
		5G networks are spreading the virus.			✓		
		Hot temperatures kill the virus.			✓		
		Vitamin C is an effective treatment.			✓		
		Ibuprofen exacerbates COVID-19.			✓		
		The flu shot provides immunity to COVID-19.			✓		
		Hydroxychloroquine is an effective treatment.			✓		
		UV rays kill the virus.			✓		
		There is a cure/vaccine for COVID-19.			✓		
		Parcels from China can spread the virus.			✓		
		The virus causing COVID-19 was engineered and released from a Chinese laboratory in Wuhan.			✓		
**Explanatory variables**							
	**Digital health literacy^d^**						
		I know what health resources are available on the internet.	✓				
		I know where to find helpful health resources on the internet.	✓				
		I know how to find helpful health resources on the internet.	✓				
		I know how to use the internet to answer my questions about health.	✓				
		I know how to use the health information I find on the internet to help me.	✓				
		I have the skills I need to evaluate the health resources I find on the internet.	✓				
		I can tell high-quality health resources from low-quality health resources on the internet.	✓				
		I feel confident in using information from the internet to make health decisions.	✓				
	**Perceived threat of COVID-19^e^**						
		Perceived public threat of COVID-19 (scale: 1=no threat at all to 10=very serious public health threat)	✓		✓		
		Perceived likelihood of personally getting sick from COVID-19 (scale: 1=not at all to 5=I definitely will)	✓		✓		
	**Confidence in the government^f^**						
		I am confident in the information about COVID-19 provided by the government.	✓		✓		
		I am satisfied with the amount of information about COVID-19 provided by the government.	✓		✓		
		I follow government advice on social distancing to help protect the wider community.	✓		✓		
		I am concerned that government recommendations about COVID-19 are not safe, or not enough is being done.	✓		✓		
	**Trust in institutions^g^**						
		Scientists involved in developing and testing new ways to control COVID-19	✓				
		Researchers involved in tracking and predicting COVID-19 cases	✓				
		Medical institutions (general practitioners, hospitals) involved in managing COVID-19 cases	✓				
	**COVID-19 information sources: social media**						
		Social media reported as being used as a top-3 information source	✓		✓		
	**Rejection of official accounts^h^**						
		Much of the information we receive is wrong.			✓		
		I often disagree with commonly held views about the world.			✓		
		Official government accounts of events cannot be trusted.			✓		
		Major events are not always what they seem.			✓		

^a^Four items, adapted from validated vaccine conspiracy beliefs scale [19]; scale: 1=strongly disagree to 7=strongly agree.

^b^This question was from a validated scale and referred to vaccines in general, not a COVID-19 vaccine.

^c^Ten items, taken from Australian Government Myth busting website [13]; scale: 1=definitely false to 5=definitely true.

^d^Mean of 8 items from the eHealth Literacy Scale (eHeals) [20]; scale: 1=Strongly disagree to 5=Strongly agree.

^e^Two individual items, adapted from [21].

^f^Mean of 4 items, adapted from national Australian survey on vaccination [22]; scale: 1=strongly disagree to 7=strongly agree.

^g^Mean of 3 items adapted from [23]; scale: 1=strongly disagree to 7=strongly agree.

^h^Mean of 4 items, adapted from [24]; scale: 1=strongly disagree to 5=strongly agree.

### Statistical Analysis

Analyses were conducted using Stata/IC (v16.1; StataCorp LLC). The threshold for statistical significance was set at *P*<.05. Descriptive statistics (means and SD for continuous variables, and frequency and relative frequency for categorical variables) were calculated for participant characteristics and study outcomes. To reduce the number of outcomes for analysis, misinformation beliefs at baseline were combined into a single measure using principal component analysis (PCA). Associations between the extracted misinformation component and possible explanatory variables were explored using truncated linear regression (with lower-bound truncation based on the minimum numerically possible value of the extracted misinformation component that would result from responding “strongly disagree” to all question items included in the PCA) controlling for sociodemographic factors previously shown to be associated with misinformation beliefs [17].

Changes in misinformation beliefs across study rounds were examined using linear mixed models with random intercepts by the participant and robust standard errors. These items were analyzed individually owing to changes in the items included in each round.

Dimension reduction using PCA was applied to the 10 specific COVID-19 myth items (included in Round 3 of the study). Multivariable truncated regression models (with lower-bound truncation as described above) were used to examine associations with the extracted components, using the same explanatory variables as for the analysis of misinformation beliefs from Round 1. Where survey items were repeated in Round 3 (ie, perceived threat of COVID-19, confidence in government, and use of social media as a “top-3” information source), this version of the variable was included; otherwise, the response at baseline was carried forward (ie, digital health literacy, institutional trust, and sociodemographic variables). An additional explanatory variable added in Round 3 (ie, rejection of official accounts) was also included in these models.

## Results

### Sample Characteristics (Cross-Sectional and Longitudinal)

Sample characteristics by each month are summarized in Table 2. When compared to national data, our sample was slightly older, included more females, had higher educational attainment, and was less likely to speak a LOTE at home.

**Table 2:** Sample characteristics by study round (1-3).

Sample description			Values						
			Cross-sectional		Longitudinal				
			April: Round 1 (n=4362)		April: Round 1 (n=2006)^a^		May: Round 2 (n=1882)		June: Round 3 (n=1369)
Age (years), mean (SD)			42.6 (17.4)		43.1 (16.6)		43.0 (16.6)		44.6 (16.7)
**Gender, n (%)**									
	Male	1698 (39.9)		635 (31.7)		589 (31.3)		433 (31.6)	
	Female	2615 (60)		1338 (66.7)		1263 (67.1)		911 (66.5)	
	Not specified/other	49 (1.1)		33 (1.6)		30 (1.6)		25 (1.8)	
**Education, n (%)**									
	High school or lower	934 (21.4)		317 (15.8)		302 (16.1)		198 (14.5)	
	Certificate I-IV^b^	617 (14.1)		223 (11.1)		204 (10.8)		140 (10.2)	
	University education	2811 (64.4)		1466 (73.1)		1378 (73.1)		1031 (75.3)	
**Language other than English spoken at home,** **n (%)**			274 (6.3)		75 (3.7)		70 (3.7)		51 (3.7)
	Cantonese	31 (0.7)		8 (0.4)		8 (0.4)		4 (0.3)	
	Mandarin	28 (0.6)		12 (0.6)		11 (0.6)		2 (0.1)	
	Spanish	19 (0.4)		6 (0.3)		6 (0.3)		2 (0.1)	
	Vietnamese	15 (0.3)		6 (0.3)		5 (0.3)		4 (0.3)	
	Hindi	14 (0.3)		1 (<0.1)		1 (0.1)		1 (0.1)	
	Arabic	11 (0.3)		1 (<0.1)		1 (0.1)		0	
	Indonesian	10 (0.2)		4 (0.2)		4 (0.2)		1 (0.1)	
	Urdu	10 (0.2)		2 (0.1)		2 (0.1)		2 (0.1)	
	Other^c^	136 (3.1)		35 (1.7)		32 (1.7)		35 (2.6)	
Socioeconomic status quintile^d^, mean (SD)			3.6 (1.4)		3.7 (1.4)		3.7 (1.4)		3.7 (1.4)
**Residential location, n (%)**									
	New South Wales	2001 (45.9)		1025 (51.1)		964 (51.2)		719 (52.5)	
	Victoria	788 (18.1)		323 (16.1)		303 (16.1)		201 (14.7)	
	Queensland	672 (15.4)		280 (14.0)		254 (13.5)		183 (13.4)	
	Western Australia	371 (8.5)		138 (6.9)		133 (7.1)		91 (6.6)	
	South Australia	238 (5.5)		93 (4.6)		89 (4.7)		64 (4.7)	
	Tasmania	144 (3.3)		79 (3.9)		74 (3.9)		58 (4.2)	
	Australian Capital Territory	120 (2.8)		62 (3.1)		59 (3.1)		49 (3.6)	
	Northern Territory	28 (0.6)		6 (0.3)		6 (0.3)		4 (0.3)	

^a^Round 1 longitudinal sample is a subsample of those included in the cross-sectional Round 1 sample, and who responded to at least one follow-up survey. This group was recruited via social media only.

^b^Certificates I-IV are tertiary qualifications; see Australian Qualifications Framework [25].

^c^Languages other than English spoken at home with cell counts <10 at baseline.

^d^Socioeconomic Indexes for Areas and Index of Relative Socioeconomic Advantage and Disadvantage Quintiles [1-5] based on participants’ residential postcode.

### Misinformation Beliefs and Associations with Sociodemographic, Cognitive, and Psychosocial Variables (Cross-Sectional Sample in April)

One month into lockdown in Australia, of the 4362 participants, 753 (17.3%) agreed that data about the effectiveness of vaccines is often made up (this survey question referred to vaccines in general, not a COVID-19 vaccine); 652 (15%) agreed that herd immunity would be beneficial for COVID-19, but this is covered-up; 603 (13.8%) agreed that the threat of COVID-19 is greatly exaggerated; and 595 (13.6%) agreed that the Australian government restrictions are stronger than required. Responses on these items were moderately correlated (pairwise *r* was between 0.36 and 0.63), with good internal consistency (Cronbach α=.78) and sufficient sampling adequacy (Kaiser-Meyer-Olkin or KMO=0.76). PCA of these items resulted in the extraction of a single component with an eigenvalue greater than 1, accounting for 60.7% of the variance (component loadings are provided in Table S1 in Multimedia Appendix 1). Estimated marginal mean values from the multivariable regression model of misinformation beliefs at baseline are provided in Table 3. Stronger agreement with misinformation beliefs was significantly associated with younger age, male gender, lower education, and primarily speaking a LOTE at home (*P*<.001 for all). After controlling for these variables, misinformation beliefs were found to be significantly associated (*P*<.001 for all) with lower levels of digital health literacy, perceived threat of COVID-19, confidence in the government, and trust in scientific institutions.

**Table 3:** Multivariable truncated linear regression of strength of agreement with misinformation beliefs at Round 1^a^. Higher values of the outcome indicate greater support for misinformation.

Explanatory variables				Value		Estimated marginal mean differences (95% CIs)		*P* value
**Sociodemographic variables**								
	Age in years, mean (SD)			42.5 (17.4)		−0.023 (−0.028, −0.018)		<.001
	Female gender (vs male)^b^, n (%)			2568 (59.9)		−0.384 (−0.541, −0.226)		<.001
	**Education (vs high school or less), n (%)**							<.001
		Certificate I-IV^c^	609 (14.2)		0.114 (−0.133, 0.360)		.37	
		University education	2760 (64.4)		−0.270 (−0.459, −0.080)		.005	
	Language other than English spoken at home, n (%)			270 (6.3)		0.847 (0.569, 1.126)		<.001
	Socioeconomic status quintile, mean (SD)			3.60 (1.40)		−0.050 (−0.105, 0.005)		.08
**Additional explanatory variables**								
	Digital health literacy^d^, mean (SD)			4.04 (0.74)		−0.250 (−0.356, −0.144)		<.001
	Perceived public threat of COVID-19^e^, mean (SD)			7.64 (2.17)		−0.336 (−0.372, −0.300)		<.001
	Not likely to get sick, n (%)			1091 (25.5)		0.649 (0.475, 0.823)		<.001
	Confidence in government^f^, mean (SD)			5.15 (1.06)		−0.143 (−0.222, −0.063)		<.001
	Institutional trust^g^, mean (SD)			5.95 (1.06)		−0.663 (−0.738, −0.587)		<.001
	Social media used as a top-3 information source, n (%)			1923 (44.9)		0.151 (−0.001, 0.307)		.06

^a^Sample for analysis comprised 4286 complete records; occasional instances of missing data for explanatory variables were not imputed due to the small proportion of missingness (76/4362, 1.8%).

^b^Marginal mean differences are not reported for gender reported as “not specified” or “other” due to small sample size, but this data was included in the regression model.

^c^Certificates I-IV are tertiary qualifications; see Australian Qualifications Framework [25].

^d^Mean of 8 items, range: 1-5.

^e^Likert scale, range: 1-10.

^f^Mean of 4 items, range: 1-7.

^g^Mean of 3 items, range: 1-7.

### Changes in Misinformation Beliefs Over Time (Longitudinal Sample in April-June)

**Figure 1:**
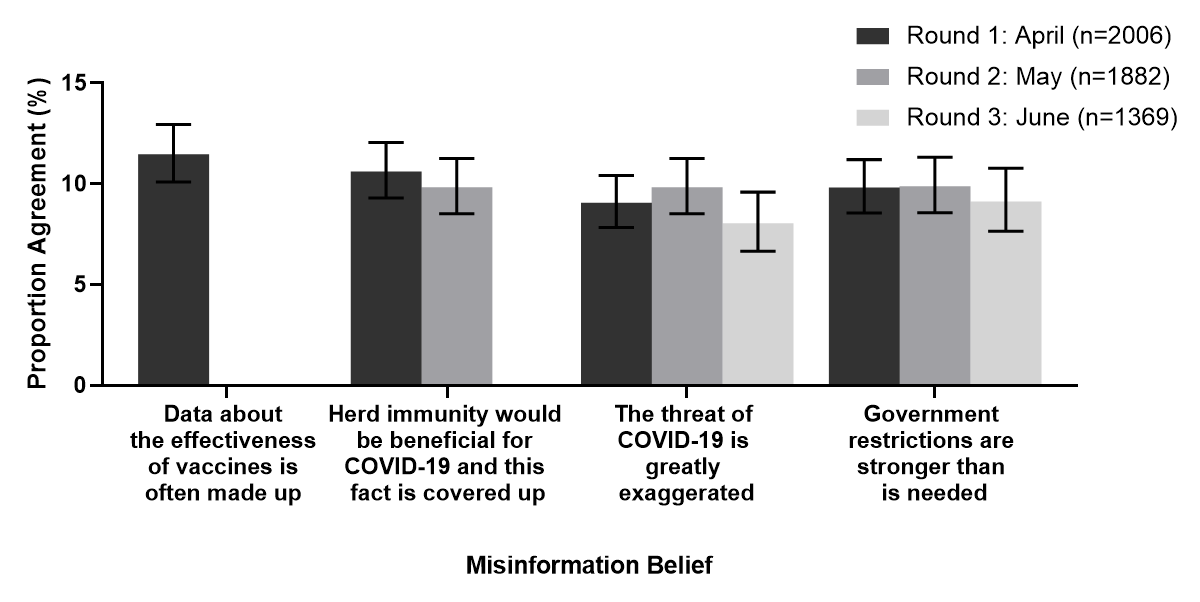
Prevalence of agreement (i.e., responding as somewhat agree (5) to strongly agree (7) on the 1 to 7 Likert scale) with misinformation beliefs by study month. Error bars indicate 95% confidence intervals.].

The prevalence of agreement with misinformation beliefs across the study period is shown in Figure 1, which appears to be generally consistent over time. Estimated mean values from the fixed portion of linear mixed models are presented in Table 4. A significant effect of time (*P*=.006) was identified for the misinformation belief that the threat of COVID-19 is greatly exaggerated, with pairwise contrasts showing an increase in this belief between April and May; however, this difference was not maintained in June. There was a decrease in the belief that herd immunity is beneficial for COVID-19 but is covered up between April and May (*P*<.001). No difference was observed in across the study period with regard to the strength of government restrictions belief (*P*=.41).

**Table 4:** Estimated means (95% CIs) of fixed effects from linear mixed models analyses (with random intercepts by the participant) of agreement with misinformation beliefs by study month and estimated mean differences (95% CIs) for pairwise comparisons to Round 1 (April).

Misinformation belief (scale range: 1-7)	April: Round 1 (n=2006)	May: Round 2 (n=1882)			June: Round 3 (n=1369)			
	Mean (95% CIs)	Mean (95% CIs)	Mean difference^a^ (95% CIs)	*P* value		Mean (95% CIs)	Mean difference^a^ (95% CIs)	*P* value
Data about the effectiveness of vaccines is often made up	2.37 (2.30, 2.44)	N/A^b^	N/A	N/A		N/A	N/A	N/A
Herd immunity would be beneficial for COVID-19 and this fact is covered up	2.52 (2.46, 2.59)	2.39 (2.32, 2.46)	−0.13 (−0.19, −0.07)	<.001		N/A	N/A	N/A
The threat of COVID-19 is greatly exaggerated	1.99 (1.93, 2.05)	2.07 (2.01, 2.14)	0.08 (0.03, 0.13)	.002		2.04 (1.98, 2.10)	0.05 (−0.01, 0.10)	.11
Government restrictions are stronger than is needed	2.14 (2.08, 2.21)	2.16 (2.09, 2.22)	0.01 (−0.04, 0.07)	.67		2.19 (2.12, 2.25)	0.04 (−0.02, 0.11)	.19

^a^Mean difference compared to Round 1 completed in April 2020.

^b^N/A: not applicable.

### Specific Misinformation Beliefs and Associations With Sociodemographic, Cognitive, and Psychosocial Variables (Longitudinal Sample In June)

The level of agreement across the 10 COVID-19 misinformation items from the Australian Government website had moderate internal consistency (Cronbach α=.693) and sufficient sampling adequacy (KMO =0.761). Application of PCA (with varimax rotation) identified a 3-component solution with eigenvalues greater than 1, which cumulatively accounted for 51.15% of the variance (see Table S2 in Multimedia Appendix 1 for component loading and proportion agreement with each item). Examination of the contributing items to each component resulted in the following 3 labels:

1. Symptom management and prevention misinformation: principal component (PC)1 (explaining 18.9% of the total variance)
2. Causes and transmission misinformation: PC2 (explaining 16.7% of the total variance)
3. Immunity and cure misinformation: PC3 (explaining 15.6% of the total variance)

Regarding specific misinformation concerning symptom management and prevention, of the 1369 participants in Round 3, 301 (22%) participants agreed that hot temperatures kill the virus, 295 (21.5%) participants agreed that UV rays kill the virus, and 179 (13.1%) participants agreed that ibuprofen exacerbates COVID-19 (see Table S2 in Multimedia Appendix 1). Greater support for symptom management and prevention misinformation (PC1) was significantly associated with younger age and male gender, as well as with lower institutional trust and greater rejection of official accounts (PC1) after controlling for demographics (age, gender, education, and LOTE; see Table 5). For misinformation regarding causes and transmission, of the 1369 participants, 167 (12.2%) participants agreed that the virus causing COVID-19 was engineered and released from a Chinese laboratory in Wuhan, 57 (4.2%) participants agreed that parcels from China could spread the virus, and only 8 (0.6%) participants agreed that 5G networks are responsible for the spread of the virus. Causes and transmission misinformation (PC2) was significantly associated with less education and more social disadvantage. Greater belief in these statements was also associated with lower digital health literacy, reduced perceived public threat, reduced institutional trust, and greater rejection of official accounts after controlling for sociodemographic variables (PC2; see Table 5). Regarding misinformation about immunity and cure, of the 1369 participants in the sample, 62 (4.5%) participants agreed that vitamin C is an effective treatment, 55 (4%) participants agreed that there is a cure or vaccine for COVID-19, 32 (2.3%) participants agreed that hydroxychloroquine is an effective treatment, and 15 (1.1%) participants agreed that the flu shot provides immunity. Greater support for immunity and cure misinformation (PC3) was significantly associated with younger age. After controlling for sociodemographic factors, lower digital health literacy, reduced perceived public threat, reduced institutional trust, and greater rejection of official accounts were associated with greater belief in these statements (PC3; see Table 5).

**Table 5:** Multivariable truncated linear regression of the misinformation beliefs in June (Round 3)^a^. Higher values of the outcome indicate greater support for these beliefs. Data are presented as estimated marginal mean differences (95% confidence intervals) and *P* values.

Explanatory Variable			Value	Estimated marginal mean differences (95% CIs) and *P* values								
				Symptom management and prevention (PC1)	*P* value	Causes and transmission (PC2)	*P* value	Immunity and cure (PC3)	*P* value			
**Sociodemographic variables^b^**												
	Age in years, mean (SD)		44.6 (16.7)	−0.007 (−0.014, −0.001)	.03	0.005 (−0.003, 0.014)	.23	−0.021 (−0.029, −0.013)	<.001			
	Female gender (vs male)^c^, n (%)		909 (66.5)	−0.397 (−0.610, −0.184)	<.001	0.222 (−0.087, 0.530)	.16	−0.088 (−0.341, 0.165)	.49			
	**Education (vs high school or less), n (%)**				.51		<.001		.25			
		Certificate I-IV^d^	140 (10.2)	0.155 (−0.245, 0.556)	.45	0.401 (−0.109, 0.912)	.13	0.266 (−0.185, 0.716)	.25			
		University education	1028 (75.3)	0.173 (−0.121, 0.467)	.25	−0.498 (−0.899, −0.096)	.02	−0.051 (−0.388, 0.285)	.77			
	Language other than English spoken at home, n (%)		51 (3.7)	−0.298 (−0.827, 0.230)	.27	0.463 (−0.254, 1.18)	.21	0.212 (−0.375, 0.799)	.48			
	Socioeconomic status quintile, mean (SD)		3.69 (1.39)	−0.032 (−0.104, 0.040)	.01	−0.212 (−0.313, −0.111)	<.001	−0.007 (−0.092, 0.079)	.88			
**Additional explanatory variables**												
	Digital health literacy^b,e^, mean (SD)		4.18 (0.67)	0.105 (−0.046, 0.255)	.17	−0.304 (−0.512, −0.097)	.004	−0.444 (−0.618, −0.270)	<.001			
	Perceived public threat of COVID-19^f^, mean (SD)		7.33 (2.44)	−0.027 (−0.069, 0.016)	.22	−0.074 (−0.133, −0.015)	.01	−0.057 (−0.107, −0.007)	.03			
	Not likely to get sick, n (%)		123 (9.0)	0.083 (−0.264, 0.429)	.64	−0.285 (−0.781, 0.211)	.26	0.133 (−0.267, 0.535)	.52			
	Confidence in government^g^, mean (SD)		5.52 (0.94)	0.028 (−0.094, 0.149)	.67	0.117 (−0.054, 0.288)	.18	0.051 (−0.093, 0.194)	.49			
	Institutional trust^b,h^, mean (SD)		6.15 (0.95)	−0.229 (−0.339, −0.119)	<.001	−0.599 (−0.750, −0.448)	<.001	−0.226 (−0.353, −0.099)	<.001			
	Social media used as a top-3 information source, n (%)		680 (49.8)	0.107 (−0.094, 0.307)	.30	0.200 (−0.087, 0.486)	.17	0.177 (−0.063, 0.417)	.15			
	Rejection of official accounts^i^, mean (SD)		2.36 (0.83)	0.172 (0.031, 0.313)	.02	0.451 (0.245, 0.657)	<.001	0.337 (0.169, 0.506)	<.001			

^a^Sample for analysis (n=1366); occasional cases of missing data for explanatory variables were not imputed due to a small proportion of missingness (3/1369, 0.2%).

^b^Values obtained in April (Round 1) and carried forward.

^c^Marginal mean differences are not reported for gender reported as “not specified” or “other” owing to small sample size but were included in the regression model.

^d^Certificates I-IV are tertiary qualifications; see the Australian Qualifications Framework [25].

^e^Mean of 8 items, range: 1-5.

^f^Likert scale, range: 1-10.

^g^Mean of 4 items, range: 1-7.

^h^Mean of 3 items, range: 1-7.

^i^Mean of 4 items, range: 1-5.

## Discussion

### Principal Findings

Our analysis showed lower institutional trust, lower digital health literacy, and greater rejection of official accounts were associated with a stronger agreement with COVID-19 misinformation beliefs. Misinformation was also more common among participants who primarily spoke a LOTE at home, in younger age groups, and in males. The most commonly held misinformation beliefs were concerning symptom management and prevention. We found small changes between April and May in two of the misinformation items: an increase in agreement with “COVID-19 is greatly exaggerated” and a decrease in agreement with “herd immunity is beneficial for COVID-19 but is covered up.” Despite these differences being statistically significant, they likely have little to no practical importance (ie, only a 0.08- and 0.12-unit change, respectively, on a 7-point scale). Notably, the proportion of participants agreeing with each item remained generally consistent over time during and after lockdown restrictions.

The agreement rates of COVID-19 misinformation beliefs were lower than those reported in other countries [26,27], but we note that our study was not sampled to be representative of the Australian population. An Australian poll conducted in May 2020 found relatively high support (12%-77%) for misinformation beliefs relating to the creation, spread, and prevention of the virus [28]. Interestingly, compared with the results of this poll, we found a much lower prevalence of people agreeing that 5G networks are spreading the virus. The poll found demographic patterns similar to our findings, wherein male and younger participants agreed with a range of COVID-19 misinformation beliefs more than other groups. Studies have shown that in the United States and the United Kingdom, younger people are more likely to hold conspiracy beliefs about COVID-19 [29,30]. Moreover, other studies have found that American men are more likely to agree with COVID-19 conspiracy theories than women [31].

The association between misinformation beliefs and lower education, LOTE, younger age, and male gender point toward important gaps in public health messaging to these specific groups. Our recent study highlights similar disparities in knowledge and behavior [17], as well as issues with the complexity of government health information about COVID-19. People with less education and LOTE had a poorer understanding of COVID-19 symptoms and were less frequently able to identify behaviors to prevent infection. Recently, attention has been focused on the importance of reaching people who do not speak English as their first language [32]. Our study further highlights the need for health information to be written to meet diverse health literacy requirements and targeted to specific study groups. For instance, young people and representatives of culturally and linguistically diverse groups should be involved in the design of COVID-19 messages to ensure appropriate tonality and delivery of the message. This can be achieved by testing communications with these groups, running consumer focus groups before releasing messages to the public, and ensuring representation on public health communication teams [33]. Ideally, a coproduction approach should be used to ensure targeted community messages about COVID-19 prevention are relevant and effective.

The provision of quality information online is unlikely to be a sufficient strategy to counter the influence of misinformation if digital health literacy is not accounted for. Messaging and debunking must be delivered on multiple trusted channels [34], consistent in content and style, and conveyed in local languages to ensure engagement with all communities [35]. Emerging evidence supports the idea that psychological inoculation—pre-emptively exposing people to small doses of misinformation techniques—can build resistance to false information across cultures [36]. It will be important to invest in programs teaching digital health literacy and healthy skepticism of health news, including interventions nudging people to consider the accuracy of COVID-19–related news content before sharing it further [37]. Finally, partnerships between public health authorities and trusted organizations to deliver information and correct misinformation should be utilized where possible [38]. Corrective messages are most successful when they offer a coherent explanation for how and why a belief based on misinformation is incorrect [39]. Research shows that corrective information can counter misperceptions and improve belief accuracy after an individual has been exposed to misinformation [40].

Timely, accurate, and transparent messaging is vital to gaining public trust in communication from authorities ahead of other, less credible sources [41]. Although there now is intense global interest aimed at limiting the spread of misinformation in the first place [2,36,42], this will require “a sustained and coordinated effort by independent fact-checkers, independent news media, platform companies, and public authorities to help the public understand and navigate the pandemic” [43].

Around the world and in Australia, antilockdown protests have taken place in capital cities, with protesters voicing opposition to vaccination, telecommunication towers, and COVID-19 hoax. Researchers have recently investigated the degree to which misinformation about COVID-19 is associated with people’s willingness to adhere to public health recommendations and government-enforced measures; they found that willingness decreases significantly as the strength of misbeliefs increases [44,45]; this also includes decreased intentions to avail a COVID-19 vaccination [46]. In some cases, misinformation has led to serious harm, such as the Iranian methanol poisoning episode [47]. The spread of misinformation is an ongoing area of concern as Australia and other countries worldwide continue to live with the fluctuating realities of a global pandemic. Correcting misinformation should be viewed as a vitally important science and health policy activity [48]. Importantly, the more extreme conspiracy beliefs were rare; for example, fewer than 1% of the participants in our study sample endorsed the 5G conspiracy. However, other beliefs were held by over 20% of the participants in certain demographics, indicating widespread confusion or simply outdated information spread among people, such as that regarding the use of ibuprofen.

### Strengths and Limitations

The study was large and diverse but not representative of the national population. Given this, caution is needed in generalizing from these prevalence findings. The sample was recruited via an online panel and social media. The majority of participants were well educated and a low proportion were from culturally and linguistically diverse groups. Therefore, this sample may not represent the demographics of all people concerned by COVID-19 and vulnerable to misinformation, including older adults. Participants recruited via Dynata were not included in the follow-up (ie, Rounds 2 and 3) due to funding constraints. Moreover, details of the specific social media platform(s) used by the participants (eg, YouTube, Twitter, and Facebook) were not captured in our survey, but it is important to note that both good- and poor-quality information may be obtained through these channels. (Mis)information can come from various sources such as family and friends, television, radio, print media, or misinformed health care providers (including primary, allied, alternative, and complementary health sectors). The use of social media as a “top-3” information source was comparable across education categories (ie, 45% for all 3 categories); however, given the abovementioned limitation, it is unclear which platform is being used by whom.

The longitudinal design of this study enabled us to evaluate whether misinformation beliefs changed over the course of the pandemic. By design, the survey items changed across time; however, this prevented us from being able to determine longitudinal changes in the PC derived at the baseline. Finally, some of the misinformation items are likely contextual and subjective (eg, “the government restrictions are stronger than is needed”), which may have influenced the interpretation and responses of some participants.

Incorrect information about COVID-19—whether labeled as misinformation, myth, conspiracy theory, or rumor—circulates every day, and our knowledge regarding the value of various preventive interventions has progressed during the course of the pandemic. While we acknowledge that some of the misinformation items included in this survey were subject to legitimate inquiry (eg, advice recommending against the use of ibuprofen was issued by the World Health Organization early in the pandemic but then retracted), they have since been demonstrated to be scientifically incorrect, classified as misinformation, and included on myth-busting lists of leading public health institutions. The broader implication is that the groups identified in this study are more likely to agree with misinformation, including younger age, male, lower education, lower health literacy, and LOTE, may not be receiving up-to-date, evidence-based advice.

### Conclusions

Misinformation can undermine public health efforts. The findings of this survey-based study highlight important gaps in communication effectiveness in the context of the COVID-19 pandemic. In efforts to prebunk and debunk misinformation, public health authorities must urgently build new partnerships with trusted, influential stakeholders and social media companies to reach the groups identified in this study. Communicators must pay close attention to ensuring that all communities can access, understand, and act on reliable COVID-19 advice.

## Appendix

Multimedia Appendix 1 Principal component loadings from principal component analyses for COVID-19 misinformation beliefs at (1) Round 1 and (2) Round 3 with percentage agreement and descriptive statistics presented.

## Abbreviations

KMO: Kaiser-Meyer-Olkin
LOTE: language other than English
PC: principal component
PCA: principal component analysis

## References

[1] Tan ASL, Lee C, Chae J (2015). Exposure to health (mis)information: lagged effects on young adults' health behaviors and potential pathways. J Commun.

[2] Zarocostas J (2020). How to fight an infodemic. The Lancet.

[3] Depoux A, Martin S, Karafillakis E, Preet Raman, Wilder-Smith A, Larson H (2020). The pandemic of social media panic travels faster than the COVID-19 outbreak. J Travel Med.

[4] Kouzy R, Abi Jaoude Joseph, Kraitem Afif, El Alam Molly B, Karam Basil, Adib Elio, Zarka Jabra, Traboulsi Cindy, Akl Elie W, Baddour Khalil (2020). Coronavirus goes viral: quantifying the COVID-19 misinformation epidemic on Twitter. Cureus.

[5] Mian A, Khan S (2020). Coronavirus: the spread of misinformation. BMC Med.

[6] (2020). Novel Coronavirus (2019-nCoV) Situation Report - 13. World Health Organization.

[7] Krause NM, Freiling I, Beets B, Brossard D (2020). Fact-checking as risk communication: the multi-layered risk of misinformation in times of COVID-19. Journal of Risk Research.

[8] Vosoughi S, Roy D, Aral S (2018). The spread of true and false news online. Science.

[9] Feemster KA, Szipszky C (2020). Resurgence of measles in the United States: how did we get here?. Curr Opin Pediatr.

[10] Hall V, Banerjee E, Kenyon C, Strain A, Griffith J, Como-Sabetti K, Heath J, Bahta L, Martin K, McMahon M, Johnson D, Roddy M, Dunn D, Ehresmann K (2017). Measles outbreak - Minnesota April-May 2017. MMWR Morb Mortal Wkly Rep.

[11] Ruijs WL, Hautvast JL, van der Velden K, de Vos S, Knippenberg H, Hulscher ME (2011). Religious subgroups influencing vaccination coverage in the Dutch Bible belt: an ecological study. BMC Public Health.

[12] Jama A, Ali M, Lindstrand A, Butler R, Kulane A (2018). Perspectives on the measles, mumps and rubella vaccination among Somali mothers in Stockholm. Int J Environ Res Public Health.

[13] (2020). COVID-19 Mythbusting. Australian Government.

[14] Scheufele DA, Krause NM (2019). Science audiences, misinformation, and fake news. Proc Natl Acad Sci U S A.

[15] Sheeran P, Maki A, Montanaro E, Avishai-Yitshak A, Bryan A, Klein WMP, Miles E, Rothman AJ (2016). The impact of changing attitudes, norms, and self-efficacy on health-related intentions and behavior: A meta-analysis. Health Psychol.

[16] Lewandowsky S, Cook J, Ecker UKH (2020). Under the Hood of The Debunking Handbook 2020: A consensus-based handbook of recommendations for correcting or preventing misinformation. Climate Social Science Network.

[17] McCaffery K, Dodd Rachael H, Cvejic Erin, Ayrek Julie, Batcup Carys, Isautier Jennifer Mj, Copp Tessa, Bonner Carissa, Pickles Kristen, Nickel Brooke, Dakin Thomas, Cornell Samuel, Wolf Michael S (2020). Health literacy and disparities in COVID-19-related knowledge, attitudes, beliefs and behaviours in Australia. Public Health Res Pract.

[18] Dodd RH, Cvejic E, Bonner C, Pickles K, McCaffery KJ, Sydney Health Literacy Lab COVID-19 group (2020). Willingness to vaccinate against COVID-19 in Australia. Lancet Infect Dis.

[19] Shapiro GK, Holding A, Perez S, Amsel R, Rosberger Z (2016). Validation of the vaccine conspiracy beliefs scale. Papillomavirus Res.

[20] Norman CD, Skinner HA (2006). eHEALS: The eHealth Literacy Scale. J Med Internet Res.

[21] Wolf M, Serper Marina, Opsasnick Lauren, O'Conor Rachel M, Curtis Laura, Benavente Julia Yoshino, Wismer Guisselle, Batio Stephanie, Eifler Morgan, Zheng Pauline, Russell Andrea, Arvanitis Marina, Ladner Daniela, Kwasny Mary, Persell Stephen D, Rowe Theresa, Linder Jeffrey A, Bailey Stacy C (2020). Awareness, attitudes, and actions related to COVID-19 among adults with chronic conditions at the onset of the U.S. outbreak: a cross-sectional survey. Ann Intern Med.

[22] My Chow, Danchin Margie, Willaby Harold W, Pemberton Sonya, Leask Julie (2017). Parental attitudes, beliefs, behaviours and concerns towards childhood vaccinations in Australia: A national online survey. Aust Fam Physician.

[23] Frew PM, Murden R, Mehta CC, Chamberlain AT, Hinman AR, Nowak G, Mendel J, Aikin A, Randall LA, Hargreaves AL, Omer SB, Orenstein WA, Bednarczyk RA (2019). Development of a US trust measure to assess and monitor parental confidence in the vaccine system. Vaccine.

[24] Uscinski JE, Enders AM, Klofstad C, Seelig M, Funchion J, Everett C, Wuchty S, Premaratne K, Murthi M (2020). Why do people believe COVID-19 conspiracy theories?. HKS Misinfo Review.

[25] AQF qualifications. Australian Qualifications Framework.

[26] Douglas KM, Uscinski JE, Sutton RM, Cichocka A, Nefes T, Ang CS, Deravi F (2019). Understanding Conspiracy Theories. Political Psychology.

[27] Geldsetzer P (2020). Knowledge and Perceptions of COVID-19 Among the General Public in the United States and the United Kingdom: A Cross-sectional Online Survey. Ann Intern Med.

[28] (2020). Belief in Conspiracy Theories. Essential Research.

[29] Allington D, Duffy B, Wessely S, Dhavan N, Rubin J (2020). Health-protective behaviour, social media usage and conspiracy belief during the COVID-19 public health emergency. Psychol Med.

[30] Schaeffer K (2020). Nearly three-in-ten Americans believe COVID-19 was made in a lab. Pew Research Centre.

[31] Cassese EC, Farhart CE, Miller JM (2020). Gender differences in COVID-19 conspiracy theory beliefs. Pol & Gen.

[32] Grey A (2020). Australia's multilingual communities are missing out on vital coronavirus information. ABC News: The Conversation.

[33] (2018). The best research is produced when researchers and communities work together. Nature.

[34] Fridman I, Lucas Nicole, Henke Debra, Zigler Christina K (2020). Association between public knowledge about COVID-19, trust in information sources, and adherence to social distancing: cross-sectional survey. JMIR Public Health Surveill.

[35] Ghio D, Lawes-Wickwar S, Tang MY, Epton T, Howlett N, Jenkinson E, Stanescu S, Westbrook J, Kassianos A, Watson D, Sutherland L, Stanulewicz N, Guest E, Scanlan D, Carr N, Chater A, Hotham S, Thorneloe R, Armitage C, Arden M, Hart J, Byrne-Davis L, Keyworth C What influences people's responses to public health messages for managing risks and preventing disease during public health crises? A rapid review of the evidence and recommendations. PsyArXiv.

[36] Roozenbeek J, van der Linden S, Nygren T (2020). Prebunking interventions based on the psychological theory of “inoculation” can reduce susceptibility to misinformation across cultures. HKS Misinfo Review.

[37] Pennycook G, McPhetres Jonathon, Zhang Yunhao, Lu Jackson G, Rand David G (2020). Fighting COVID-19 misinformation on social media: experimental evidence for a scalable accuracy-nudge intervention. Psychol Sci.

[38] Bavel Jay J Van, Baicker Katherine, Boggio Paulo S, Capraro Valerio, Cichocka Aleksandra, Cikara Mina, Crockett Molly J, Crum Alia J, Douglas Karen M, Druckman James N, Drury John, Dube Oeindrila, Ellemers Naomi, Finkel Eli J, Fowler James H, Gelfand Michele, Han Shihui, Haslam S Alexander, Jetten Jolanda, Kitayama Shinobu, Mobbs Dean, Napper Lucy E, Packer Dominic J, Pennycook Gordon, Peters Ellen, Petty Richard E, Rand David G, Reicher Stephen D, Schnall Simone, Shariff Azim, Skitka Linda J, Smith Sandra Susan, Sunstein Cass R, Tabri Nassim, Tucker Joshua A, Linden Sander van der, Lange Paul van, Weeden Kim A, Wohl Michael J A, Zaki Jamil, Zion Sean R, Willer Robb (2020). Using social and behavioural science to support COVID-19 pandemic response. Nat Hum Behav.

[39] Walter N, Murphy ST (2018). How to unring the bell: A meta-analytic approach to correction of misinformation. Communication Monographs.

[40] van der Meer TGLA, Jin Y (2020). Seeking formula for misinformation treatment in public health crises: The effects of corrective information type and source. Health Commun.

[41] Legido-Quigley H, Asgari N, Teo YY, Leung GM, Oshitani H, Fukuda K, Cook AR, Hsu LY, Shibuya K, Heymann D (2020). Are high-performing health systems resilient against the COVID-19 epidemic?. The Lancet.

[42] Stephenson J (2020). United Nations seeks to counter COVID-19 misinformation with digital first responders. JAMA Health Forum.

[43] Brennen JS, Simon F, Howard PN, Nielsen RK (2020). Types, sources, and claims of COVID-19 misinformation. Reuters Institute.

[44] Imhoff R, Bruder M (2020). Speaking (un–)truth to power: conspiracy mentality as a generalised political attitude. Eur J Pers.

[45] Oliver JE, Wood T (2014). Medical conspiracy theories and health behaviors in the United States. JAMA Intern Med.

[46] Teovanović P, Lukić P, Zupan Z, Lazić A, Ninković M, Žeželj I (2020). Irrational beliefs differentially predict adherence to guidelines and pseudoscientific practices during the COVID‐19 pandemic. Appl Cognit Psychol.

[47] Delirrad M, Mohammadi Ali Banagozar (2020). New methanol poisoning outbreaks in Iran following COVID-19 pandemic. Alcohol Alcohol.

[48] Caulfield T Does debunking work? Correcting COVID-19 misinformation on social media. OSF.

