# Supplementary material for: COVID-19 Misinformation Trends in Australia: Prospective Longitudinal National Survey
Source: J Med Internet Res. 2021 Jan 7;23(1):e23805. doi: 10.2196/23805 (PMC7800906; doi:10.2196/23805)
Supplement: Multimedia Appendix 1 [file jmir_v23i1e23805_app1.docx]

**Table S1.** Misinformation beliefs at baseline (Round 1) PC loading.

| Item | Component Loading |
| --- | --- |
| Data about the effectiveness of vaccines is often made up | 0.429 |
| The threat of COVID-19 is greatly exaggerated | 0.546 |
| Herd immunity would be beneficial for COVID-19 and this fact is covered up | 0.497 |
| Australian government restrictions are stronger than is needed | 0.521 |

**Table S2**. Agreement^a^ and summary statistics for COVID-19 misinformation items (selected from the Australian Government COVID-19 Mythbusting website) at Round 3 (n=1369); and descriptive statistics and component loadings^b^ from Principal Component Analysis].

| **Items [1 to 5]** | **Disagree, n (%)** | **Unsure, n (%)** | **Agree, n (%)** | **Mean (SD)** | **Component loadings** | | |
| --- | --- | --- | --- | --- | --- | --- | --- |
|  |  |  |  |  | **PC1** | **PC2** | **PC3** |
| 5G networks are spreading the virus | 1328 (96.9) | 356 (2.5) | 8 (0.6) | 1.10 (0.43) |  | 0.590 |  |
| Hot temperatures kill the virus | 808 (58.9) | 262 (19.1) | 301 (22.0) | 2.30 (1.28) | 0.596 |  |  |
| Vitamin C is an effective treatment | 1128 (82.3) | 181 (13.2) | 62 (4.5) | 1.62 (0.90) |  |  |  |
| Ibuprofen exacerbates COVID-19 | 746 (54.4) | 446 (32.5) | 179 (13.1) | 2.33 (1.07) | 0.351 |  |  |
| The flu shot provides immunity to COVID-19 | 1317 (96.1) | 39 (2.8) | 15 (1.1) | 1.17 (0.51) |  |  | 0.658 |
| Hydroxychloroquine is an effective treatment | 1008 (73.5) | 331 (24.1) | 32 (2.3) | 1.85 (0.87) |  |  |  |
| UV rays kill the virus | 734 (53.5) | 342 (25.0) | 295 (21.5) | 2.39 (1.25) | 0.618 |  |  |
| There is a cure/vaccine for COVID-19 | 1218 (88.8) | 98 (7.2) | 55 (4.0) | 1.37 (0.81) |  |  | 0.629 |
| Parcels from China can spread the virus | 1179 (86.0) | 135 (9.8) | 57 (4.2) | 1.66 (0.82) |  | 0.460 |  |
| The COVID-19 virus was engineered and released from a Chinese laboratory in Wuhan | 1008 (73.5) | 196 (14.3) | 167 (12.2) | 1.95 (1.09) |  | 0.620 |  |

^a^Items were recoded to disagree [1-2], neutral/unknown [3], agree [4-5].

^b^Component loadings less than |0.3| have been omitted.
